# Sex Dependent Disparities in the Central Innate Immune Response after Moderate Spinal Cord Contusion in Rat

**DOI:** 10.3390/cells13070645

**Published:** 2024-04-06

**Authors:** Mousumi Ghosh, Jinyoung Lee, Ashley N. Burke, Thomas A. Strong, Jacqueline Sagen, Damien D. Pearse

**Affiliations:** 1 The Miami Project to Cure Paralysis, University of Miami Miller School of Medicine, Miami, FL 33136, USA; jinsoobyul0309@gmail.com (J.L.); ashburke95@gmail.com (A.N.B.); tas156@med.miami.edu (T.A.S.); jsagen@med.miami.edu (J.S.); dpearse@med.miami.edu (D.D.P.); 2 The Department of Neurological Surgery, University of Miami Miller School of Medicine, Miami, FL 33136, USA; 3 Department of Veterans Affairs, Veterans Affairs Medical Center, Miami, FL 33136, USA; 4 The Neuroscience Program, University of Miami Miller School of Medicine, Miami, FL 33136, USA; 5 The Interdisciplinary Stem Cell Institute, University of Miami Miller School of Medicine, Miami, FL 33136, USA

**Keywords:** spinal cord injury, microglia, macrophages, sex, inflammation, neuroprotection, NFκB pathway, p38 kinase, iNOS, ARG1

## Abstract

Subacute spinal cord injury (SCI) displays a complex pathophysiology associated with pro-inflammation and ensuing tissue damage. Microglia, the resident innate immune cells of the CNS, in concert with infiltrating macrophages, are the primary contributors to SCI-induced inflammation. However, subpopulations of activated microglia can also possess immunomodulatory activities that are essential for tissue remodeling and repair, including the production of anti-inflammatory cytokines and growth factors that are vital for SCI recovery. Recently, reports have provided convincing evidence that sex-dependent differences exist in how microglia function during CNS pathologies and the extent to which these cells contribute to neurorepair and endogenous recovery. Herein we employed flow cytometry and immunohistochemical methods to characterize the phenotype and population dynamics of activated innate immune cells within the injured spinal cord of age-matched male and female rats within the first week (7 days) following thoracic SCI contusion. This assessment included the analysis of pro- and anti-inflammatory markers, as well as the expression of critical immunomodulatory kinases, including P38 MAPK, and transcription factors, such as NFκB, which play pivotal roles in injury-induced inflammation. We demonstrate that activated microglia from the injured spinal cord of female rats exhibited a significantly diminutive pro-inflammatory response, but enhanced anti-inflammatory activity compared to males. These changes included lower levels of iNOS and TLR4 expression but increased levels of ARG-1 and CD68 in females after SCI. The altered expression of these markers is indicative of a disparate secretome between the microglia of males and females after SCI and that the female microglia possesses higher phagocytic capabilities (increased CD68). The examination of immunoregulatory kinases and transcription factors revealed that female microglia had higher levels of phosphorylated P38^Thr180/Tyr182^ MAPK and nuclear NFκB pp50^Ser337^ but lower amounts of nuclear NFκB pp65^Ser536^, suggestive of an attenuated pro-inflammatory phenotype in females compared to males after SCI. Collectively, this work provides novel insight into some of the sex disparities that exist in the innate immune response after SCI and indicates that sex is an important variable when designing and testing new therapeutic interventions or interpretating positive or negative responses to an intervention.

## 1. Introduction

Spinal cord injury (SCI) is a devastating neurological condition with significant and lifelong consequences due to the inability of the CNS to undergo vigorous self-repair. Though some progress has been made in the identification of promising therapies in experimental paradigms, a dearth of protective or restorative therapies exists clinically. One important biological variable that influences endogenous recovery after SCI is sex [1,2,3,4,5]. The previous work in our [1] and other [4,5] laboratories provided evidence that following SCI in rodents, sex differences existed in outcomes in which females exhibited improved locomotor recovery and greater preservation of both white and gray matter compared to males [1]. In two subsequent studies, female rodents showed reduced lesion sizes and faster sensory recovery after SCI [6] or displayed a lower neuroinflammatory response, improved locomotor function, better cognitive performance, and less depressive-like behaviors than age-matched male animals, a finding that was more pronounced when SCI occurred at a young adult age [7]. Within the human SCI patient population, the lower incidence of injury among females limits the acquisition of adequate clinical data for comparing the extent of functional recovery or prognosis of SCI between sexes. The previous studies by Sipski et al. [3] have suggested that sex disparities exist regarding neurological outcomes according to the level of injury. Further investigation is necessary to elucidate how findings from animal models can be translated to human spinal cord injury (SCI), particularly in terms of sex differences and therapeutic implications. Understanding the relationship between cell responses and outcomes in animal models versus clinical data in human SCI patients, especially concerning sex differences, is crucial. Exploring sex-dependent variations could provide valuable insights for designing sex-specific therapeutic interventions.

One critical CNS cell response to injury is that of the microglial cell. In addition to the effect of sex on microglial cell behavior in SCI [7,8], many recent reports have highlighted the critical role it plays in microglia function during normal CNS development [9,10,11,12,13] and amid a myriad of neurological conditions [14,15,16,17,18,19,20,21]. Improvements in the recovery of females after SCI has been ascribed, in part, to sexual dimorphism that exists in the response of microglia to injury, including their phenotype, secretome, and thus, functional role [7,8,22,23]. In a recent study by Stewart et al. [8], significant sex-related differences in the transcriptional and inflammatory profiles of microglia isolated after subacute SCI were observed. Microglia derived from female mice were more active and showed an increase in reactive oxygen species (ROS)-producing genes, such as NADPH-Oxidase (NOX2) and CYBB, as well as tissue remodeling MRC1 (CD206), whereas microglia from males exhibited increased expression of the complement protein, C1qa. C1qa is known for its important immunomodulatory role in mediating the downregulation of the pro-inflammatory cytokines IL-1β and IL-6 in macrophages through the suppression of NFκB signaling [24]. In a study carried out by Li et al. [7], an opposite dichotomy in microglia function was observed after SCI in which microglia from injured male mice displayed higher activation and increased expression of pro-inflammatory cytokines IL-1β and TNF-α compared to females. Despite the contrasting results of these investigations in ascribing greater pro-inflammatory roles to either male or female microglia after SCI, the findings point to the fact that pronounced sex-related disparities do exist in microglial responses to CNS injury, and further investigation is warranted to understand these differences as they could significantly affect how individuals recover after SCI and respond to protective or restorative therapies clinically. In the present investigation, we sought to examine if sex disparities existed within activated microglia after SCI in the regulation of enzymes, receptors, kinases, and transcription factors that are central to injury-induced neuroinflammation.

Inducible nitric oxide synthase (iNOS) and Arginase-1 (ARG1) are well established markers for evaluating the phenotypic state of these immune cells [25], sharing an enzyme substrate and playing dichotomous roles in modulating the immune response to insults. iNOS is associated with pro-inflammatory microglia and macrophages, contributing to the production of reactive nitric oxide that leads to oxidative stress and consequent damage to cells and tissues. Conversely, ARG1 is linked to anti-inflammatory polarization and contributes to tissue repair and remodeling.

Another important molecule that is expressed in microglia and has emerged as a crucial player in neuroinflammatory responses is Toll-like receptor 4 (TLR4) [26]. TLR4 is a crucial pattern recognition receptor involved in modulation of the innate immune response post-SCI [27,28]. The prior research has shown that selective TLR4 deletion alters cytokine and extracellular matrix expression after SCI, leading to decreased secondary damage and enhanced functional recovery [29]. Herein we evaluated sex disparities in TLR4 after SCI.

Downstream of these receptor systems and enzymes in microglia involved in the initial sensing of the injury are kinases and transcription factors that drive gene expression programs, which govern the type of behavior and function the microglial cell performs in response to the changes occurring in its extracellular environment. In this investigation, we sought to determine whether sex disparities existed within microglia after SCI in the regulation of immunomodulatory kinases and transcription factors such as P38 MAP kinase [30] and NFκB subunits p65 and p50, which stand as pivotal regulators of pro-inflammatory genes [31,32], exerting profound influence on pathophysiological cascades encompassing tissue damage, neurological impairment, and the genesis of neuropathic pain subsequent to SCI. The selection of P38 MAP kinase and NFκB subunits p65 and p50 as the focus of our investigation is based on a robust rationale derived from their well-established roles in SCI pathophysiology [33,34] and their potential sex-specific effects [35]. P38 MAP kinase is a crucial signaling molecule involved in the regulation of inflammation, apoptosis, and cellular responses to stress, all of which are prevalent following SCI. Numerous studies have implicated P38 MAP kinase activation in exacerbating secondary injury cascades, contributing to neuronal cell death and impairing functional recovery following SCI [33]. Furthermore, the emerging evidence suggests that P38 MAP kinase activity may exhibit sex-specific variations in response to other neurological conditions [36], both from a deleterious and protective point of view [30,36], potentially influencing the differential outcomes observed between males and females [35]. Similarly, NFκB signaling, mediated by its subunits p65 and p50, plays a pivotal role in orchestrating the inflammatory response and consequent secondary tissue damage following SCI. Activation of NFκB induces the transcription of pro-inflammatory cytokines and mediators [31], exacerbating tissue damage and neuroinflammation in the injured spinal cord. However, little information is available to determine if sex-related differences correlate to NFκB signaling, especially in SCI, and the observed disparities in functional outcomes between males and females.

This study stems from findings of the prior investigations and work from our laboratory identifying significant sex-related differences in SCI outcomes, with females often exhibiting superior recovery compared to males. Here, we aimed to identify the potential role that key pro-inflammatory molecules, like iNOS and TLR4, immunomodulatory kinases such as pP38 MAPK, and components of the signaling pathway associated with the crucial transcription factor NFκB, played in mediating sex-specific responses to SCI and their implications for therapeutic intervention. The present study highlights novel sex disparities in the expression and subcellular distribution of these crucial molecules in activated microglia after experimental SCI in the rat. It is envisioned that through elucidating how sex influences the functional and phenotypic state of reactive microglia and subsequent recovery processes, we will better comprehend the factors driving disparate outcomes among males and females. By considering the implications of sex-specific differences in microglial function and inflammatory responses in rodent models of SCI, this study will provide knowledge that is crucial for developing targeted therapeutic interventions tailored to the unique needs of both sexes, thereby improving overall treatment efficacy and clinical outcome in SCI patients.

## 2. Materials and Methods

### 2.1. Reagents

The different primary antibodies; their hosts, sources, and dilutions employed for immunohistochemistry; and flow cytometry analysis are provided in Table 1.

### 2.2. Animals

Age matched (16–17 weeks old), equal numbers of adult male and female Fischer rats (Charles River Company, *n* = 32; 180–200 g) were housed according to NIH and The Guide for the Care and Use of Animals. All the animal procedures employed in this study were approved by the Institutional Animal Care and Use Committee (IACUC) of the University of Miami.

### 2.3. Pre-Operative Preparation

Before surgical procedures, the animals were weighed and anesthesia induced by a combination of 2% isoflurane and 30% oxygen. Anesthesia adequacy was determined by monitoring the respiratory rate, corneal reflexes, and pedal reflexes. The back of the animals was then shaved and aseptically wiped with chlorhexidine (Phoenix Pharmaceutical Inc., St. Joseph, MO, USA). To prevent dryness during the surgical procedure, artificial tears ointment (Rugby, Livonia, MI, USA) was applied to the eyes. Vaginal impedance in female rats was examined using the Model MK-11 Rat Vaginal Impedance Checker (Muromachi Kikai, Tokyo, Japan) to determine if the rodents were within the 4-day period of their estrous cycle. If they were within this period they were not used. Before gently inserting the probe into the deepest point of the vagina, the probe was lubricated with non-spermicidal sterile lubricating jelly (First Priority, Elgin, IL, USA). The rats were positioned on a homeothermic blanket system (Harvard Apparatus Ltd., Kent, UK) to ensure a stable body temperature of 37 ± 0.5 °C was maintained through a feedback monitoring system with rectal probe throughout the surgical procedure. The female rats underwent spinal cord contusion following confirmation that they were outside of the 4–5 day window of the estrus cycle, with the aim to prevent the impact of rapid hormonal fluctuations during this period [37], which would modulate the neuroinflammatory response post-SCI.

### 2.4. Moderate Thoracic Spinal Cord Contusion Injury and Post-Operative Care

SCI was induced using The Multicenter Animal Spinal Cord Injury Study (MASCIS) impactor, following the previously described procedures [38]. In brief, the dorsal surface of the thoracic level spinal cord was exposed without disrupting the dura by a laminectomy at the thoracic vertebra level T8. A moderate SCI was induced by releasing a 10.0 g rod from a height of 25.0 mm onto the exposed region of the spinal cord as described previously [38]. Injury parameters, including contusion impact height, velocity, and compression, were documented. The animals with height or velocity errors exceeding 7% or with a compression distance outside of the range of 1.75 to 2.25 mm were excluded from the study. After surgery, the muscles were sutured in layers and the skin was closed with metal wound clips. The animals recovered from anesthesia in a warmed cage and were then transferred to another cage, as pairs, with ad libitum access to food and water, Alpha Dri^®^ bedding (changed three times a week, Fort Worth, TX, USA), and water bottles with long curved sipper tubes for easy access. Daily post-operative care [27] involved Gentamicin (5 mg/kg, intramuscular; Abbott Laboratories, North Chicago, IL, USA) administered immediately after surgery and continued daily for seven days. Moderate–severe SCI in the rat results in the loss of the ability of animals to voluntarily void urine, a state that makes them prone to urinary tract infections, which, if not prophylactically-treated post-SCI with Gentamycin for a prolonged period of 7 days, can be a major contributor to acute mortality [39]. The analgesic Buprenex (0.03 mg/kg, subcutaneous; Reckitt Benckiser, Richmond, VA, USA) was given immediately post-surgery and then continued daily for an additional two days. Lactated Ringers (5 cc, subcutaneous) was administered twice a day for seven days or longer based on the animals’ hydration status. Following moderate–severe thoracic SCI, rats initially lose their ability to void their bladders and require manual emptying of the bladder twice a day for up to a period of approximately 7 days or until return of normal bladder function. Therefore the bladders of both male and female rats were manually expressed twice daily using gentle compression of the abdominopelvic region (Crede method) until normal bladder function returned. The study groups comprised eight SCI rats of each sex with half used for flow cytometry and half for immunohistochemistry (IHC). Matching cohorts of naïve male and female rats were also used for comparison.

### 2.5. Animal Perfusion and Tissue Extraction

At the survival endpoint of 7 days after SCI contusion or immediately for naïve, the animals were perfused. The rats were euthanized by carbon dioxide inhalation and then subjected to transcardial perfusion. After perfusion with 500 mL cold (4 °C) physiological saline, animals were perfused with 400 mL cold phosphate-buffered 4% paraformaldehyde (0.1M, pH 7.4). The CNS tissue was extracted and underwent overnight post-fixation in 4% paraformaldehyde, followed by cryoprotection in sucrose according to the previously described procedures [38,40]. A 2 cm segment of the thoracic T7-9 spinal cord, encompassing the injury epicenter, was embedded in M-1 embedding matrix (ThermoScientific, Kalamazoo, MI, USA) for cryosectioning into 20 series at a thickness of 20 μm (coronal) using a Leica CM3050S Cryostat (Leica Microsystems Inc., Buffalo Grove, IL, USA). The tissue sections were stored at −20 °C until further processing.

### 2.6. Microglia Isolation

The isolation of microglial cells was performed according to the previous methods [41,42] with the following changes. In brief, a cohort of male and female SCI rats was euthanized using CO_2_, followed by thoracotomy and transcardial perfusion with 300 mL cold saline. A 1 cm segment from the T7-9 thoracic level of the spinal cord, covering the entire lesion center, was dissected and suspended in 3 mL ice-cold Hibernate A-low Fluorescence media (BrainBits, Springfield, IL, USA). The tissue samples were initially cut into smaller pieces using fine scissors to facilitate mechanical dissociation, followed by enzymatic dissociation using trypsin (2.5 mg, Trypsin-EDTA, Sigma-Aldrich, St. Louis, MO, USA) and collagenase, (5 mg, Millipore-Sigma, Burlington, MA, USA) in 5 mL DMEM for 15 min at 37 °C. Next, the tissue underwent homogenization using a PYREX^®^ Dounce homogenizer with the addition of 2 mL dissociation medium, composed of 0.1% Papain dissolved in water (Carica papaya, EMD Millipore, Cat. No. 5125, Burlington, MA, USA). This mixture was then combined with a 1:1 ratio of DMEM/F12 and Dispase II (Dispase diluted to 5 U/mL; STEMCELL Technologies, Cat. No. 07913, Vancouver, BC, Canada) and DMEM/F12 (1:1; Gibco, Ref. No. 11330-032, Waltham, MA, USA). After thorough mixing, the samples were maintained at 37 °C for 20 min with gentle inversion 4–5 times at 5-min intervals. The samples were then suspended in 2 mL of Neutralization media (DMEM/F12 enriched with 10% heat inactivated FBS; Gibco, Ref. No. 16140-071, Waltham, MA, USA) containing 1% Pen Strep (Gibco, Ref. No. 15140-122, Waltham, MA, USA). Next, the samples were passed through a sterile 40 µm cell strainer (Falcon, Ref. No. 352340, New York, NY, USA) and combined with 3 mL SIP (a solution of 27 mL Percoll; GE Healthcare, 17-0891-01, Sigma, Burlington, MA, USA) and 3 mL HBSS (10×; Gibco, Ref. No. 14185-052, Waltham, MA, USA). The mixture was then gently layered over 70% SIP (a solution of 14 mL SIP and 4 mL HBSS (1×; Gibco, Ref. No. 14170-112, Waltham, MA, USA) in a 15 mL tube using a syringe. A gradient was then created by centrifuging the samples, 500× *g* at 18 °C for 45 min. The interphase layer located between 2–4 mL in each of the 15 mL tubes was collected and mixed with 8 mL 1× HBSS followed by further centrifugation, 500× *g* at 18 °C for 15 min. After centrifugation, the supernatant was discarded, and the cells were fixed in 200 µL 4% PFA for 20 min. This was followed by centrifugation, 1000× *g* at 18 °C for 10 min. The cell pellets were washed with DPBS (1×) (Gibco, Ref. No. 14190-144, Waltham, MA, USA) to remove any excess 4% PFA. The fixed cells comprising the purified microglial population were re-suspended in 200 µL of 1× DPBS and kept at 4 °C until used for flow cytometry.

### 2.7. Flow Cytometry

For immunostaining of purified microglia, the cells were suspended in flow cytometry staining buffer (1×) (R&D systems, Cat No. FC001, Minneapolis, MN, USA) and treated with purified mouse anti-rat CD32 (1 μg IgG per one million cells: BD Biosciences, Cat. No. 550271, San Jose, CA, USA) for 20 min at 4 °C. The samples were then centrifuged for 10 min, 1000× *g* at 4 °C, to remove the buffer. After re-suspension in BD Cytofix/Cytoperm solution (BD Biosciences, Cat. No. 51-2090KZ, San Jose, CA, USA) for 15–20 min at 4 °C, the samples were washed once with flow cytometry staining buffer. The cells were re-suspended in the flow cytometry staining buffer and incubated with fluorophore-conjugated antibodies overnight (in the dark) at 4 °C under gentle shaking. The following day, the cells were centrifuged for 10 min, 1000× *g* at 4 °C, and then washed twice with the flow cytometry staining buffer. Each sample was re-suspended in DPBS (1×) and filtered through a 40 µm strainer (Falcon, Ref. No. 352235, NY, USA) prior to undergoing flow cytometry. Unstained and single-color stained controls were prepared using UltraComp™ eBeads Compensation Beads (ThermoFisher Scientific, Cat. No. 01-2222, Waltham, MA, USA) for setting compensation.

The gating strategy employed for the selection of specific immune cell populations (resident microglia versus macrophages), where 10,000 cells were gated per sample, was based upon the forward (size) and the side scatter (granularity) of these innate immune cells. The strategy also ensured elimination of any dead cells or debris based upon the expression of markers, fluorescence tag conjugated CD11b, and the intensities of the homeostatic microglial marker CX3CR1 [43], which have been routinely employed to specifically identify activated macrophage and microglial cell populations. To further evaluate the specific phenotypic state of innate immune cell populations, selective markers that are characteristic of the pro- or the anti-inflammatory phenotype were employed and percentages of CD11b-CX3CR1 double positive microglial cells expressing these distinct markers following quadrant analysis were quantified and averaged across animals. Flow cytometric data acquisition was carried out with the LSR-Fortessa-HTS analyzer (BD Biosciences, Ref. No. 647800, San Jose, CA, USA), and the data analysis was performed using FCS Express™ version 7 software.

### 2.8. Immunohistochemistry

Immunohistochemical staining, followed with the quantitative assessment of the immunoreactivity of different markers of immune cells, was performed as previously described [38]. Briefly, slides with cryosectioned tissue were subjected to heat antigen retrieval using IHC-Tek epitope retrieval solution (IHC World Woodstock, Ellicott City, MD, USA) [38,44]. Following antigen retrieval, the sections underwent blocking in 2% bovine serum albumin (BSA) in PBS with 0.5% Triton-X100 for one hour at room temperature. Next, the sections were incubated with specific primary antibodies overnight at room temperature. After 3× washing with 1× PBS buffer containing 0.1% Tween 20, to visualize target proteins, the sections were incubated for 2 h with fluorescent-tagged specific secondary antibodies (ThermoFisher Scientific; Invitrogen). Following 3× washing with 1× PBS buffer containing 0.1% Tween 20, the slides were cover-slipped with micro cover glass (48404-453, VWR, Radnor, PA, USA) using Prolong Diamond Antifade mounting medium (P36970, Invitrogen, Carlsbad, CA, USA), then stored at 4 °C until confocal imaging.

### 2.9. Image Analysis

Images of the stained tissue sections were acquired using a confocal laser-scanning microscope (Olympus, Fluoview FV 1000, Center Valley, PA, USA). Images of immunostained sections with the injury epicenter were acquired for each of the specific markers from stained series. Images from three to four randomly chosen fields in the region of interest (ROI) within the lesion center of the injured tissue were captured for further quantitative assessment. The lesion site was characterized by the presence hypertrophied astrocytes, loss of neuronal cell bodies, and a predominance of microglia/macrophages exhibiting a typical activated morphology with a large cell body and short processes. The immunofluorescence intensity, indicating antibody reactivity for each specific target protein of interest, underwent blinded quantitative analysis using Image J 1.54d software. In the images presented, the tonal range and sharpness (smart sharpen, 0.9 pixels) of the Tiff files were standardized using Adobe Photoshop 2024 (Adobe Systems Inc., San Jose, CA, USA). Randomization was employed during both image acquisition and analysis to reduce bias and improve reliability. Thresholding settings were applied to the images prior to measurement of fluorescence intensity, and all measurements were performed in a blinded fashion by an independent analyst unaware of the image identities.

### 2.10. Statistical Analysis

The quantitative data obtained from flow cytometry assessment or measurements of the immunofluorescent intensity for the specific markers were plotted as the mean ± standard deviation of the mean (SD). The statistical analysis was performed employing an analysis of variance (ANOVA) followed by a Tukey test for multiple comparisons between the male and the female SCI or naïve rats using GraphPad Prism v7.0. (GraphPad Software, Inc., La Jolla, CA, USA). The differences were accepted as statistically significant at * *p* < 0.05, ** *p* < 0.01 or *** *p* < 0.0001. All errors are given as standard deviations of the mean.

## 3. Results

### 3.1. Analysis of Pro- and Anti-Inflammatory Markers in Male and Female after SCI

#### 3.1.1. Morphological Analysis of Iba1^+^ Microglia

Multicolor flow cytometry and immunohistochemical analysis were employed to quantify sex-specific disparities in microglial cell morphology and phenotype from the thoracic spinal cord at level T8 of uninjured naïve or at 7 days following moderate–severe SCI in rats. This time point of 7 days post SCI is included within the subacute phase post injury, which coincides with the peak of activated microglia and peripherally derived macrophages in the injured spinal cord [45], contributing to ongoing inflammation and neuronal and glial cell death. The 7 days time-point post SCI also corresponds to an intermediate time point prior to the peak of astroglial scarring, as well as the early period of the resolution of inflammation, associated with the initiation of limited axon growth and other dynamic processes associated with tissue remodeling [29].

**Figure 1:**
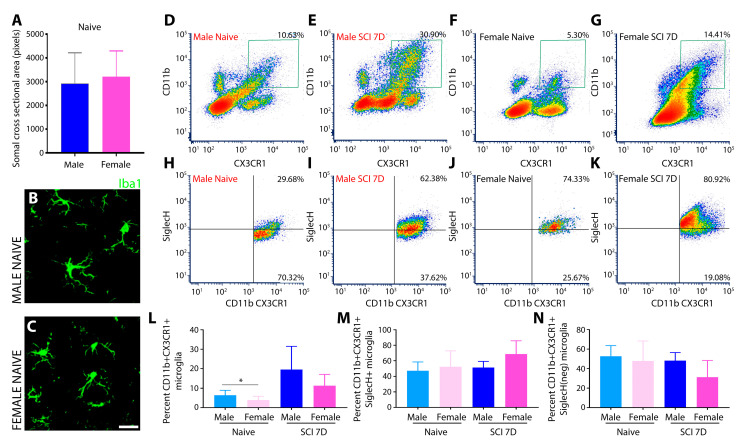
The thoracic spinal cords from male and female rodents were morphologically indifferent but exhibited significant variation with CD11b^+^CX3CR1^+^ labelled microglial cell density between the two sexes prior to SCI. No significant differences in the cellular density of CD11b^+^CX3CR1^+^ double labelled microglia or CD11b^+^CX3CR1^+^Siglec H^+^ triple labelled microglial subpopulations were observed between the two sexes post-SCI. Quantitative evaluation of microglial cell size from naïve rodents of both sexes (**A**) and representative images of Iba1+ microglia from the spinal cord of naïve male (**B**) and female rats (**C**) reveals no size or morphology differences. Scale bar = 20 μm. Morphological analysis of microglia size and cross-sectional area was conducted using Image J analysis. Data presented as mean ± SD, *n* = 50 (male microglia cell bodies) and *n* = 59 (female microglia cell bodies) were randomly quantified from biologically independent samples (*n* = 3). Representative multicolor flow cytometry density plot images show naïve and 7 days after subacute T8 SCI. The microglial cell populations were gated (gated regions are shown in green box for (**D**–**G**)) from CD11b^+^CX3CR1^+^ double labelled cells in male ((**D**,**E**), respectively) or female naïve and injured rats ((**F**,**G**), respectively) or from CD11b^+^CX3CR1^+^Siglec H^+^ triple labelled cells in naïve and SCI male ((**H**,**I**), respectively) or female rats (**J**,**K**). Representative images from biologically independent samples (male, *n* = 5; female *n* = 5) are shown. The quantitative flow cytometry analysis of the microglial population in the uninjured thoracic spinal cord of both sexes shows increased CD11b^+^CX3CR1^+^ microglial density in males (**D**,**L**) compared to that in females (**F**,**L**), but no significant differences were observed post SCI between the two sexes (**E**,**G**,**N**). Assessment of the CD11b^+^CX3CR1^+^Siglec H^+^ labelled microglial subpopulation in the naïve and the injured rodents of both sexes (**H**–**K**) showed an increased tendency of the triple labelled microglia in the lesioned T8 spinal cord of the female rodent (**K**) compared to that in the naïve female rodents (**J**) and with the naïve and the injured male counterparts (**H**,**I**) but did not exhibit statistical significance (**M**) as analyzed with a one-way ANOVA using Tukey’s multiple comparison test. No significant difference was noted for the CD11b^+^CX3CR1^+^Siglec H^+^ between the naïve and the injured spinal cord of both sexes (**N**). The data are presented as mean ± SD. In (**L**), the asterix (*) equals *p* = 0.0355 for CD11b^+^CX3CR1^+^Siglec H^+^ triple labelled microglial subpopulation comparisons between naïve male and female rodents.

To demarcate differences in microglial cell shape and size between rodents of both sexes, fixed spinal cord tissue encompassing the T8 thoracic region of the spinal cord was immunostained with Iba1 (Ionized calcium-binding adapter molecule 1), which is a characteristic microglia/macrophage-specific calcium-binding protein. The morphological analysis of Iba1^+^ immune cells involved examining confocal images taken at high magnification of randomly chosen areas of the naïve spinal cord from sections of both sexes. Randomization was applied during image acquisition and analysis to reduce bias and improve the reliability and validity of the results. Thresholding settings were adjusted before measuring fluorescence intensity. All the measurements were conducted blindly by an independent analyst unaware of the image identities. The results indicated no significant differences in overall microglial morphology, cell body size, or cross-sectional area between naïve male and female microglia (Figure 1A). Similarly, process branching, and ramification were also alike (Figure 1B,C). In images of immunostained cell preparations from the injured spinal cord, morphological assessment was inadequate to distinguish between spinal resident microglia and infiltrating macrophages using the Iba1 antibody. Hence, flow cytometry was employed, using gating strategies based on the expression of CD11b (integrin subunit alpha M involved in adhesion processes and shared by both resident CNS microglia and macrophages from the periphery) and the fractalkine receptor, CX3CR1, a homeostatic microglial marker [43], which distinguishes perivascular macrophages (CD11b^+^/CX3CR1^low^) from resident microglia (CD11b^+^/CX3CR1^high^). These markers were combined with Siglec H (sialic-acid-binding immunoglobulin-like lectins), a unique microglia-specific marker that discriminates microglia from CNS-associated macrophages and CNS-infiltrating monocytes and allows selective identification of the microglial population [46] post SCI due to highly enrichment specifically in activated microglia [47]. Flow cytometry analysis of CD11b^+^CX3CR1^+^ double labelled microglial cells indicated significantly increased microglial cell numbers in the male spinal cord compared to that of females (Figure 1D,F,L). Conversely for the injured spinal cord, males showed a trend for an increased number of activated microglia over females, though it was not statistically significant (Figure 1E,G,L). Analysis of the CD11b^+^CX3CR1^+^Siglec H^+^ triple-labeled microglial cell population showed an increased number within the lesioned cord of the females (Figure 1K) compared to that of males (Figure 1J) at 7 days after SCI, although the difference again was statistically insignificant (Figure 1N). Conversely, both the naïve and the injured spinal cord showed a tendency for an increased density of CD11b^+^CX3CR1^+^Siglec H^−^ microglia in males compared to females, but no statistically significant differences were identified between sexes (Figure 1H–K,N).

#### 3.1.2. Elevated iNOS and Decreased ARG-1 in SCI Male Compared to Female Microglia

**Figure 2:**
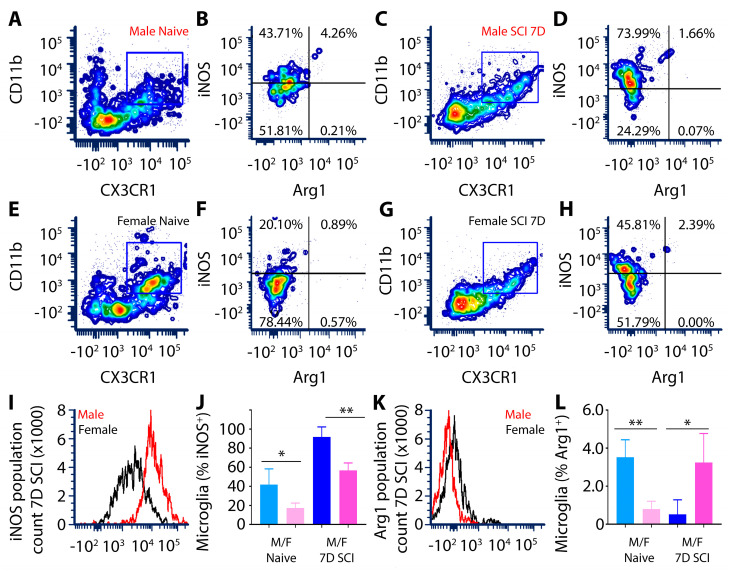
Male CD11b^+^CX3CR1^+^ microglia showed elevated iNOS and lower ARG-1 compared to females. Representative multicolor flow cytometry contour plot images and histograms of iNOS^+^ or ARG-1^+^ microglia gated from the CD11b^+^CX3CR1^+^ double labelled cell population (gated regions in (**A**,**C**,**E**,**G**) is indicated by blue box), isolated from T8 SCI or naïve rat spinal cord. Images taken from among four biologically independent samples. Microglia isolated from naïve (**A**,**B**) or SCI (**C**,**D**) male rats display significantly greater numbers of CD11b^+^CX3CR1^+^iNOS^+^ cells compared to that of female naïve (**E**,**F**) and SCI (**G**,**H**). Histograms represent comparative iNOS (**I**) and ARG-1 (**K**) CD11b^+^CX3CR1^+^ microglia count at 7 days after SCI within the injured T8 spinal cord of male versus female rats. Bar graph showing quantitative analysis of the mean fluorescence index (MFI) of iNOS (**J**) and ARG-1 (**L**) from male and female CD11b^+^CX3CR1^+^ microglia in naïve and SCI rats. Microglia from SCI male rodents exhibit significantly greater iNOS expression than their female counterparts (**I**,**J**), while for ARG-1, the converse change is observed with higher levels in SCI females compared to males (**K**,**L**). Flow cytometry data were processed using FCS Express 7 Research Edition software. Data are presented as mean ± SD with *n* = 4 biologically independent samples. In (**J**) * *p* = 0.0319 for iNOS levels in naïve male versus naïve female and ** *p* = 0.0030 for iNOS levels at 7 days post-SCI in male versus female microglia. In (**L**), ** *p* = 0.0072 for ARG-1 levels in naïve male versus female and * *p* = 0.0123 for ARG-1 levels in 7 days post-SCI in male versus female microglia using a one-way ANOVA using Tukey’s multiple comparison test. Asterisks represent significant differences.

Flow cytometry was used to measure the levels of two prominent phenotypic markers that are indicative of pro-inflammatory and anti-inflammatory microglia, respectively, inducible nitric oxide synthase (iNOS) and Arginase-1 (ARG-1), in CD11b^+^CX3CR1^+^ microglia from the naïve or T8 SCI cord of male and female rats. The selection of iNOS and ARG1 as markers for evaluating the polarization states of microglia and macrophages, respectively, was based on their well-established roles in modulating the immune response, particularly in the context of SCI. iNOS is associated with pro-inflammatory microglia/macrophages, contributing to the production of reactive nitric oxide that leads to oxidative stress and the consequent damage to cells and tissues. Conversely, ARG1 is linked to anti-inflammatory polarization and contributes to tissue repair and remodeling. The numbers of iNOS^+^ microglia in male rats were 2-fold greater than that observed in females within the naïve thoracic spinal cord (Figure 2), pointing to a possible inherent predisposition difference in pro- and anti-inflammatory responses among sexes. Additionally, the numbers of ARG1^+^ microglia were higher in uninjured male rodents than in the females within the naïve thoracic spinal cord (Figure 2).

Following T8 spinal cord contusion, the flow cytometry analysis of purified microglia derived from the injured segment showed increased iNOS expression in the double positive CD11b^+^CX3CR1^+^ activated microglial population in SCI males but a reduction in ARG1 expression compared to naïve male and SCI female (Figure 2). In contrast, the opposite response was observed in female microglia following SCI (Figure 2). A comparative histogram analysis of the fluorescence intensity of iNOS expression among sexes shows that male microglia exhibit a potent pro-inflammatory iNOS^HIGH^ARG1^LOW^ phenotype compared to that of female microglia, iNOS^LOW^ARG1^HIGH^ (Figure 2).

**Figure 3:**
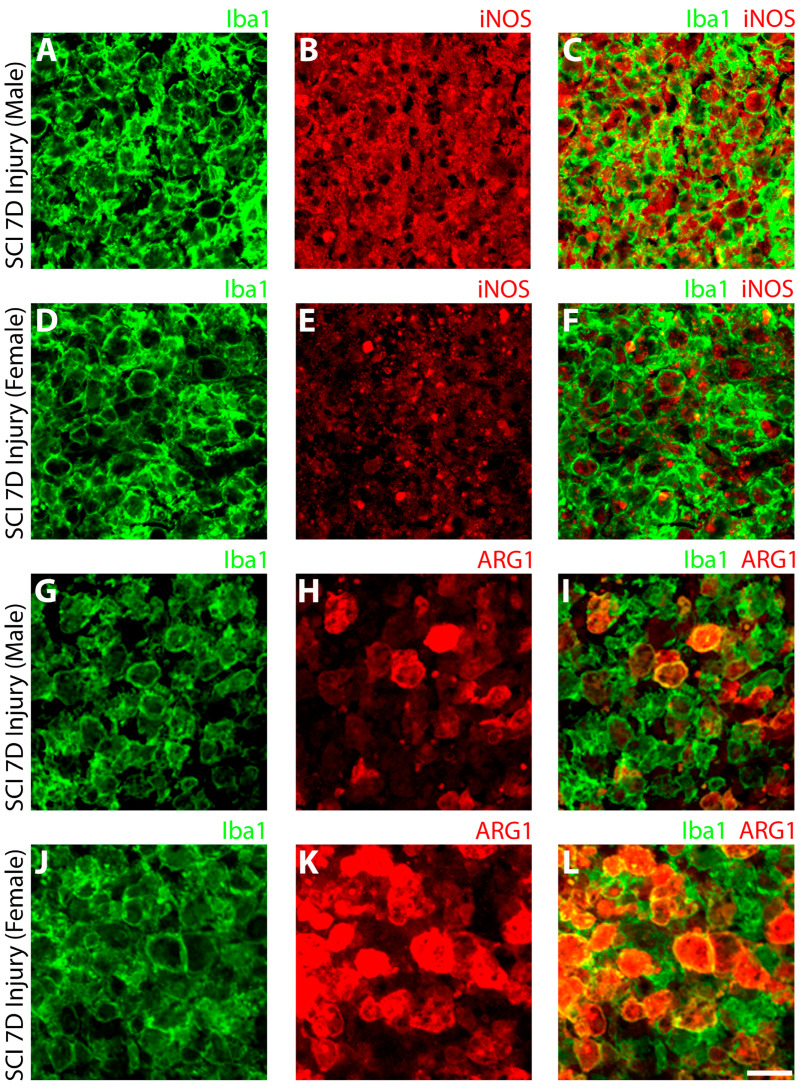
Microglia show significantly higher expression of pro-inflammatory iNOS and reduced pro-reparative ARG1 in male rats compared to females after subacute SCI. The expression of iNOS was significantly higher in Iba1^+^ microglia (and macrophages; (**A,D,G,J**)) within the T8 lesion site of male (**B**,**C**) compared to female rats (**E**,**F**). Conversely, ARG-1 expression in Iba1^+^ cells of the T8 lesion site was lower in male (**H**,**I**) compared to the female rats (**K**,**L**). Scale bar = 10 μm.

To further substantiate the findings of flow cytometry, an immunohistochemical (IHC) assessment was performed on fixed SCI tissue sections. The SCI lesion center of male rats was dominated by large numbers of Iba1^+^ innate immune cells expressing robust, cytosolic iNOS (Figure 3B,C). Although female rats also showed numerous Iba1+ innate immune cells within the SCI lesion center, iNOS expression was much lower and restricted in its cellular presence (Figure 3E,F). Examination of Iba1^+^ innate immune cells in the SCI male rats revealed only sparse expression of ARG-1 within the lesion (Figure 3H,I), whereas in female rats 7 days after SCI, many Iba1+ cells were co-stained with ARG-1 (Figure 3K,L).

#### 3.1.3. CD86 Expression in Female Microglia Is Markedly Higher Than That of Males after Subacute SCI

**Figure 4:**
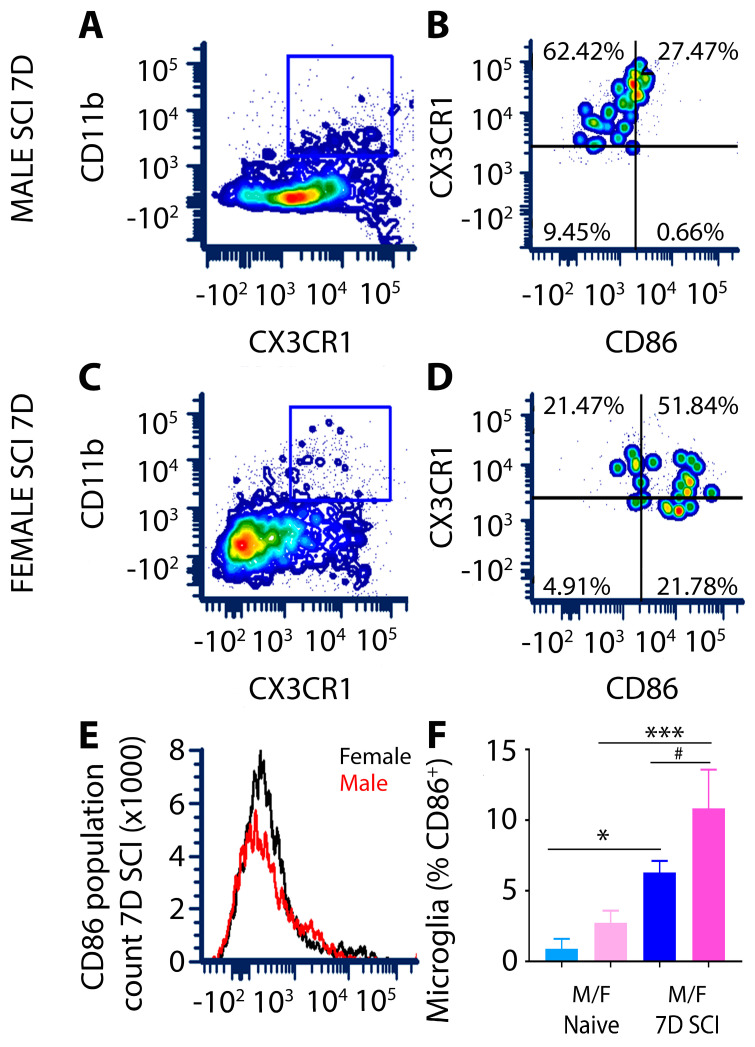
Female rats exhibit increased CD86 expression in activated microglia within the SCI lesion compared to males. Representative flow cytometry contour plot images and histograms show CD86^+^ microglia gated from a CD11b^+^CX3CR1^+^ double labelled cell population (gated regions in (**A**,**C**) is indicated by blue box) isolated from the T8 SCI or naïve spinal cord. Images taken from three biologically independent samples are shown. Microglia isolated from male (**A**,**B**) or female (**C**,**D**) rats at 7 days post-SCI showed a significant increase in the number of CD11b^+^CX3CR1^+^CD86^+^ cells in females versus males. Histogram represents comparative CD86^+^ microglia gated from the CD11b^+^CX3CR1^+^ cell population in male versus female SCI lesion sites (**E**). Bar graph illustrating the quantitation of MFI for CD11b^+^CX3CR1^+^CD86^+^ expression in male versus female microglia after subacute SCI rodents, in which female microglia from the SCI lesion site exhibit significantly higher CD86 than males (**F**). Flow cytometry data were processed using FCS Express 7 software. Data are presented as mean ± SD with *n* = 3 biologically independent samples. In (**F**) * *p* = 0.0109 for CD86^+^ levels in naïve and SCI male microglia, *** *p* = 0.0009 for CD86^+^ levels in naïve and SCI female microglia and ^#^ *p* = 0.0277 for CD86^+^ levels in male versus female microglia in the SCI lesion at 7 days using a one-way ANOVA with Tukey’s multiple comparison test. Asterisks represent significant differences.

The expression of CD86, a costimulatory factor involved in the initiation of the adaptive immune response that binds in conjunction with other co-stimulatory molecules, such as CD80, on T cells to activate them [48], was significantly increased in lesion site microglia from both males and females at 7 days after SCI compared to levels in the naïve spinal cord (Figure 4F). The flow cytometry assessment of CD86 in CD11b^+^CX3CR1^+^ double positive microglia showed that while levels of CD86 were similar among sexes in naïve animals, at 7 days after SCI, there were markedly higher numbers of CD86^+^ microglial cells within the lesion site of females compared to males (Figure 4E,F).

#### 3.1.4. Male Microglia Exhibit More Potent TLR4 Immunoreactivity Than Females after Subacute SCI

**Figure 5:**
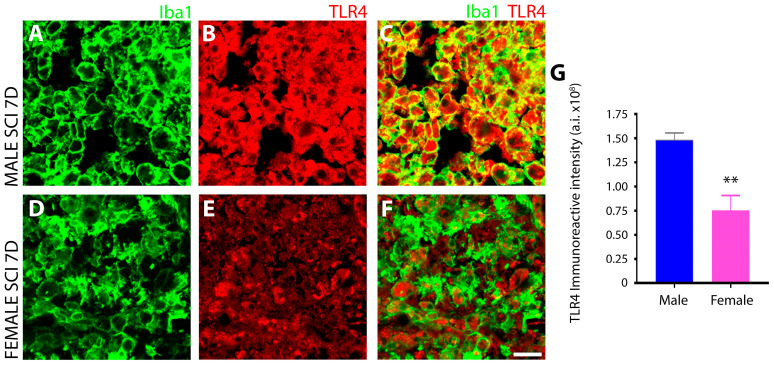
TLR4 receptor expression is significantly greater in male than female microglia after subacute SCI. TLR4 receptor expression was significantly higher in Iba1^+^ microglia (and macrophages) of the T8 spinal cord lesion site of male rats (**A**–**C**) compared to females (**D**–**F**). The bar graph (**G**) shows the quantitation of TLR4 immunoreactivity in TLR4^+^Iba1^+^ double labelled innate immune cells in male versus female SCI as measured using Image J software. Data are presented as mean ± SD from three biologically independent samples. In (**G**), ** *p* = 0.0049 for TLR4 immunoreactivity in SCI male versus female microglia using the unpaired t test. Asterisks represent significant differences. Images were acquired at 40× objective magnification with 2× digital zoom magnification. Scale bar = 10 μm.

Toll-like receptor 4 (TLR4) expression in Iba1+ microglia from the SCI lesion site was compared among sexes using IHC. Pronounced TLR4 was observed in Iba1+ microglia of the injured spinal cord of male rats at 7 days post-injury (Figure 5B,C), whereas microglia in the female counterparts displayed lower and restricted immunoreactivity that was not present in all microglial cells (Figure 5E,F). The quantitation of TLR4 fluorescent intensity in male and female Iba1+ microglia after subacute SCI revealed a two-fold greater level of TLR4 in male microglia (Figure 5G). Resting microglia in the intact spinal cord of the naïve animals exhibited basal levels showing minimal TLR4 immunoreactivity in both sexes. 

#### 3.1.5. Female SCI Rats Show Increased Expression of the Phagocytic Marker CD68 Compared to Males following Subacute SCI

**Figure 6:**
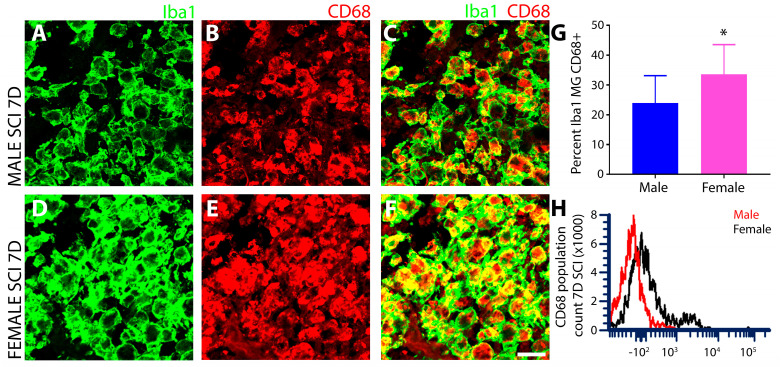
Female rodents after SCI showed increases in the phagocytic marker CD68 in the lesioned spinal cord compared to males. CD68 was significantly higher in Iba1^+^ immune cells within the SCI lesion site of female (**D**–**F**) compared to male rats (**A**–**C**), as detected by IHC. The bar graph (**G**) shows the quantitative analysis of CD68 immunoreactivity between male and female innate immune cells at 7 days after SCI, as measured using the Image J software. Data are presented as mean ± SD from three biologically independent samples. In (**G**), * *p* = 0.0472 for CD68^+^ immunoreactive intensity in Iba1+ cells from male versus female SCI analyzed using the unpaired t test. Asterisks represent significant differences. Scale bar = 10 μm. The flow cytometry histogram compares gated CD68+ phagocytic cells derived from the CD11b^+^CX3CR1^+^ microglial cell population of the injured spinal cord of male and female rats 7 days after SCI (**H**). The histogram is a representative image from three biologically independent samples.

The injury epicenter at 7 days after SCI from both sexes were analyzed for CD68 expression using IHC and flow cytometry. Double IHC for Iba1 and CD68 showed lower levels of CD68 in male (Figure 6A–C) compared to female innate immune cells (Figure 6D–F), which was further quantified and corroborated by measurements of immunoreactive intensity (Figure 6G). This would suggest that the injured spinal cord of female rodents is associated with higher phagocytic activity than those of males after subacute SCI. Flow cytometry of the CD68^+^ phagocytic cells derived from gating the CD11b^+^CX3CR1^+^ microglial population also showed greater CD68 fluorescence intensity in female versus male microglia after SCI, as pictured in the histogram analysis (Figure 6H).

#### 3.1.6. No Sex-Dependent Differences in Microglial MRC1 after Subacute SCI

**Figure 7:**
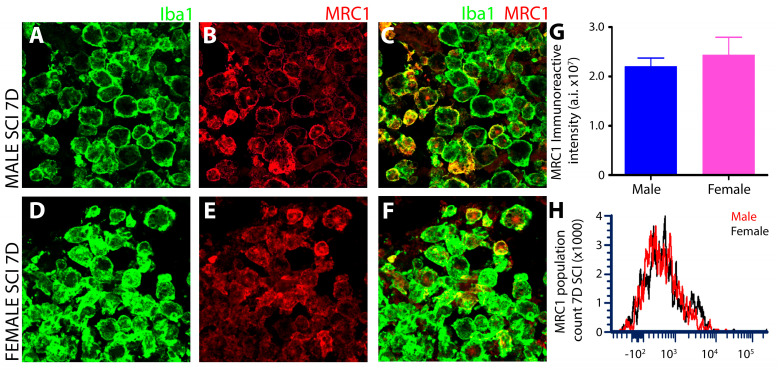
Similar levels of MRC1 are observed in male and female microglia after subacute SCI. Iba1^+^ microglia showed strong MRC1 immunoreactivity at 7 days after SCI within the injured spinal cord, but no differences existed between male (**A**–**C**) and female (**D**–**F**) microglia. The bar graph (**G**) shows quantitation of the immunoreactive intensity of MRC1 in Iba1^+^ immune cells by IHC, and the flow cytometry data similarly reveal no sex differences in MRC1 after SCI (**H**). MRC1 fluorescent intensity in Iba1^+^ cells was analyzed using Image J software. Images were acquired at 40× objective magnification with 2× digital zoom magnification. The flow cytometry histogram image shows a comparison of MRC1 in microglia among males and females that were gated from the CD11b^+^CX3CR1^+^ double labelled cell population of the SCI lesion site at 7 days (**H**). The histogram is a representative image from three biologically independent samples.

A robust expression of Mannose receptor, C type 1 (MRC1) was observed by IHC in iba1^+^ innate immune cells of the T8 injured spinal cord at 7 days post-SCI, with no differences between males and females (Figure 7A–C,G). Flow cytometry gating of MRC1^+^ microglia from the CD11b^+^CX3CR1^+^ double positive cell population revealed robust MRC1 expression post-SCI in both sexes but with no significant differences among them (Figure 7H).

### 3.2. Differential Induction and Subcellular Localization of Phosphorylated P38 MAPK among Sexes after SCI

**Figure 8:**
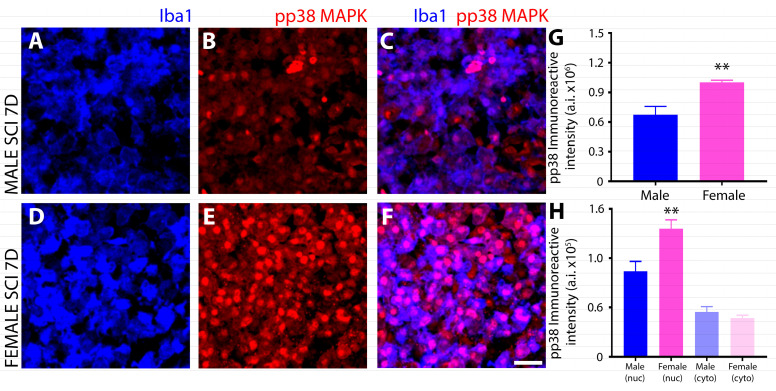
Microglial cells in female SCI rats display increased pP38 MAPK^Thr180/Tyr182^ compared to in male counterparts. Confocal images of coronal sections at the injury epicenter of the thoracic level (T8) spinal cord, after subacute spinal cord injury in both male and female rodents, were subjected to co-immunostaining using antibodies against pP38^Thr180/Tyr182^ MAPK and Iba1. Double immunofluorescence indicates that p-p38 colocalizes with Iba1 stained microglial cells in both sexes (**C**,**F**). In comparison to the males (**A**–**C**), expression of phosphorylated P38 was profoundly upregulated in the nucleus of the activated microglial cells located within the lesion site of the injured spinal cord in female rats (**D**–**F**). The bar graph (**G**) shows quantification of pP38 immunoreactivity carried out by measuring the fluorescent intensity of pP38 displayed in the Iba1^+^ cells by Image J software, which indicates a significant upregulation in female microglia compared to the male microglia. In (**G**), ** *p* = 0.0097 for percent pP38^+^ immunoreactivity in SCI male versus female Iba1^+^ microglia-macrophage population in subacutely spinal cord injured rodents was analyzed using unpaired t test. Comparative assessment of the cellular localization of pP38 in activated microglia of both sexes demonstrated differential localization with elevated levels of nuclear pP38 in female microglia compared to significantly lower levels of nuclear pP38 in male microglia, as shown in the bar graph (**H**). The percent pP38^+^ immunoreactivity in the nucleus of the Iba1^+^ microglia/macrophage population in the spinal cord injured male versus female rodents was analyzed using one way ANOVA with Tukey’s multiple comparison test where ** *p* = 0.0069. Asterisks represent significant differences. Results are expressed as mean ± SD. Images were acquired at 40× objective magnification with 2X digital zoom magnification. Scale bar = 10 µm.

IHC evaluation of the phosphorylation of P38 MAPK (P38 MAPK^Thr180/Tyr182^) in Iba1^+^ innate immune cells at 7 days after SCI showed a strong sex disparity in which female microglia exhibited a much greater, nuclear localization of the phosphorylated kinase within the injured spinal cord (Figure 8D–F) compared to males (Figure 8A–C) and as quantified by immunoreactive intensity (Figure 8G,H).

### 3.3. Sex Disparaties in the NFκB Pathway in Microglia after SCI

#### Female Microglia Show Marked Differences in the Induction and Subcellular Localization of Components of the NF-κB Signal Transduction Pathway after Subacute SCI Compared to Males

**Figure 9:**
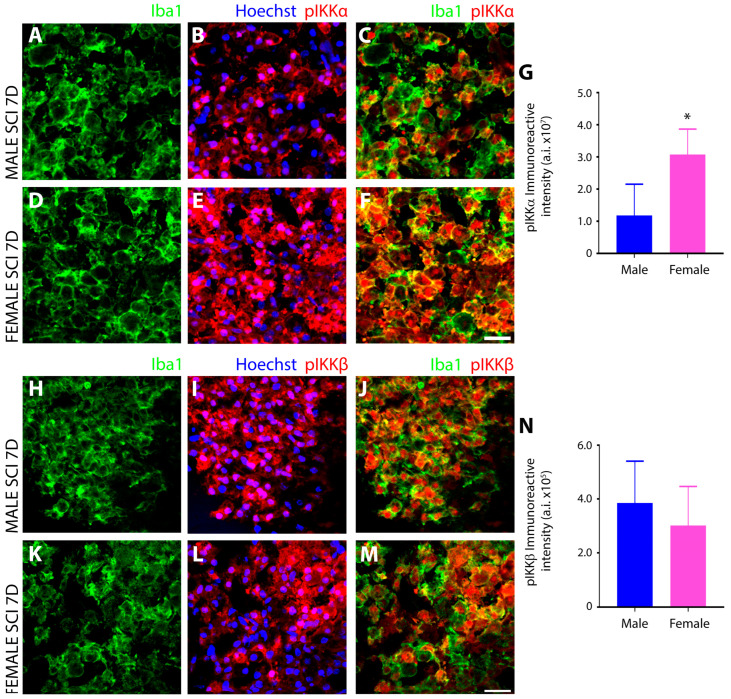
Phosphorylated IKKα^Ser176,Ser180^ was significantly greater in female microglia after SCI, whereas phosphorylated IKKβ^Tyr188^ remained unchanged among sexes. Confocal images of IHC for phospho-IKKα^Ser176,Ser180^ within the injured spinal cord 7 days after SCI shows more pronounced immunoreactivity in the iba1+ innate immune cells of females (**D**–**F**) versus males (**A**–**C**). The bar graph (**G**) quantifies these differences in pIKKα immunoreactivity as measured using Image J software. Statistical analysis using the unpaired *t* test gave * *p* = 0.0236. In comparison, phospho-IKKβ^Tyr188^ immunoreactivity in activated Iba1+ immune cells did not differ among males (**H**–**J**) and females (**K**–**M**) following subacute SCI. Quantitative assessment was performed using data from four biologically independent samples (**N**). No significant difference was seen; *p* = 0.2921 for pIKKβ^+^ immunoreactivity in Iba1+ immune cells from the injury epicenter in rodents of both sexes. Analysis was performed using the unpaired *t* test. Results are expressed as mean ± SD. Images were acquired at 40× objective magnification with 2× digital zoom magnification. Scale bar = 10 μm.

Initial IHC interrogation of the NFκB pathway targeted the evaluation of IKK phosphorylation after SCI. Serine 176 and 180 phosphorylation of IKKα (pIKKα^Ser176, Ser180^), involved in the regulation of NFκB to resolve inflammation, exhibited significantly greater levels in female (Figure 9E,F) versus male microglia (Figure 9B,C) after subacute SCI (Figure 9G), and furthermore, whereas the subcellular localization of pIKKα^Ser176, Ser180^ appeared largely nuclear in males, it was both cytoplasmic and nuclear in female Iba1^+^ innate immune cells. Conversely, tyrosine residue 188 phosphorylation of IKKβ (pIKKβ^Tyr188^), which is involved in the initiation of the inflammatory response of NFκB, was increased within the injured spinal cord to a similar extent in male and female iba1^+^ innate immune cells at 7 days after SCI (Figure 9H–N).

**Figure 10:**
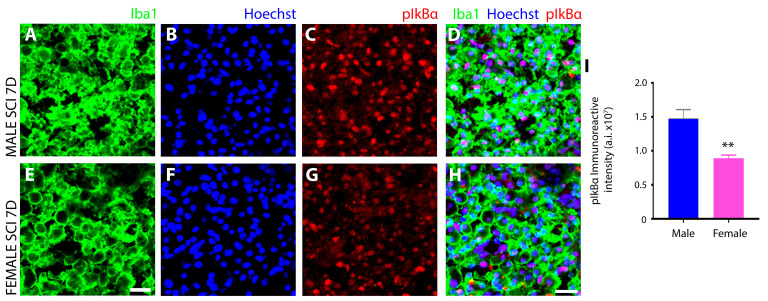
Male microglia exhibit increased expression of nuclear phosphorylated IKBα^Ser32/36^ in Iba1 innate immune cells after SCI. Iba1+ microglia (and macrophages; (**A**,**E**)) exhibited greater levels of phosphorylated pIKBα^Ser32/36^ in males (**C**,**D**) compared to females (**G**,**H**) 7 days post-SCI in rat. Cell nucleus is labelled with the nuclear dye Hoechst 33342 (**B,F**). Confocal images were acquired from random fields and the immunofluorescence intensity of pIKBα^Ser32/36^ was measured using Image J software. The bar graph (**I**) represents the quantitative assessment of pIKBα^Ser32/36^ immunoreactivity. In (**I)**, ** *p* = 0. 0.0051 for pIKBα immunoreactivity in SCI male versus female Iba1^+^ innate immune cells using the unpaired *t* test from four biologically independent samples. Scale bar = 10 μm.

Subsequently, the serine 32 and 36 residue phosphorylation of IkBα (pIkBα^Ser32/36^), a central component of classic NFκB activation and nuclear translocation, was measured by IHC. In both sexes, pIkBα^Ser32/36^ exhibited nuclear localization in iba1^+^ innate immune cells but was significantly more elevated in male (Figure 10C,D) over female microglia (Figure 10G,H) after SCI, as measured quantitatively (Figure 10I).

**Figure 11:**
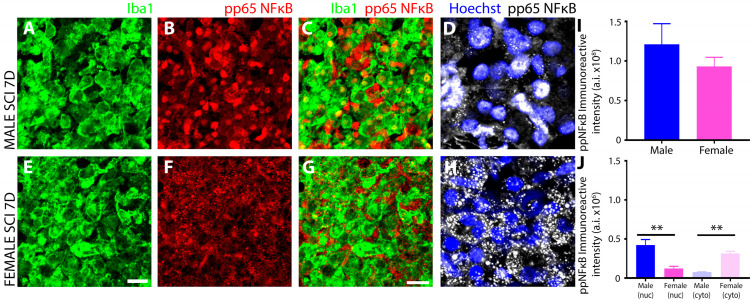
Phosphorylated NFκB pp65^Ser536^ in microglia showed sex-dependent differential levels and subcellular localization following sub-acute SCI. Iba1^+^ microglia at the SCI lesion site of male rats (**A**–**D**) displayed significantly greater nuclear translocation of phosphorylated NFκB-pp65^Ser536^ (**B**,**C**) compared to their female counterparts (**E**–**H**), in which NFκB-pp65^Ser536^ was lower in levels (**I**) and retained within the cytoplasm. The sex dependent differences in subcellular localization are quantified in graph (**J**). A *p* value = 0.3892 for the total pp65 ^Ser536^ immunoreactivity comprising of combined nuclear and cytosolic immunoreactivity in SCI male versus female Iba1^+^ innate immune cells using the unpaired t test did not show any significant difference between the two sexes. Panels (**D**,**H**) shows images at a higher magnification (180×). In (**J**), nuclear localization differences ** *p* = 0.0012 and cytosolic ** *p* = 0.0072 were analyzed using a one-way ANOVA with Tukey’s multiple comparison test. Results are expressed as mean ± SD. Images acquired at 40× objective magnification with 2× digital zoom magnification ((**A**–**C**,**E**–**G**), Scale bar = 10 μm) and at 60× objective magnification with 3× digital zoom magnification (**D**,**H**).

IHC evaluation of the phosphorylated P65 subunit of NFκB (NF-κB pP65^Ser536^), the master transactivator of inflammatory genes, revealed significant sex-related differences in levels and subcellular localization of immunoreactivity within iba1^+^ innate immune cells at 7 days after SCI within the injured segment (Figure 11). Although there was no significant difference between males and females for the overall levels of NFκB pP65 ^Ser536^ (Figure 11I), the nuclear localization levels were significantly greater than the cytoplasmic in males (Figure 11B–D,J), and the cytoplasmic levels were significantly greater than nuclear levels in females (Figure 11F–H,J).

**Figure 12:**
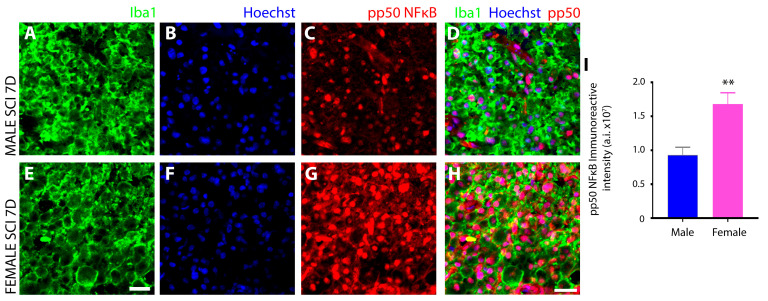
Female microglia exhibit significantly more robust nuclear expression of phosphorylated pp50 NF-κB^Ser337^ compared to males. Iba1^+^ microglia (and macrophages) exhibited significantly less nuclear phosphorylated NFκB pp50^Ser337^ within the SCI lesion in male rats (**A**–**D**) compared to that of females (**E**–**H**). The bar graph (**I**) represents quantitative assessment of the expression levels of NFκB pp50 ^Ser337^ immunoreactivity in Iba1^+^ innate immune cells. In (**I**), ** *p* = 0.0080 for NFκB pp50^Ser337^^+^ immunoreactivity in SCI male versus female Iba1^+^ immune cells, as analyzed using the unpaired *t* test. Asterisks represent significant differences. Results are expressed as mean ± SD. Images were acquired at 40× objective magnification with 2× digital zoom magnification ((**A**–**H**), Scale bar = 10 μm).

Serine residue 337 phosphorylation of the other NFκB subunit, pP50 (NF-κB pP50^Ser337^), which forms heterodimers with pP65 to promote pro-inflammatory gene expression or forms homodimers to conversely repress them, was also assessed in microglia of the injured spinal cord at 7 days after SCI. Immunoreactivity of NF-κB pP50^Ser337^ was less pronounced in iba1^+^ innate immune cells of males (Figure 12A–D) compared to females (Figure 12E–H) and as measured quantitatively after SCI (Figure 12I).

## 4. Discussion

In our study, we investigated sex-specific differences in microglial activation and signaling pathways following SCI. We observed significant disparities between male and female rats in the activation of microglia, as well as in the expression levels of key proteins involved in inflammatory signaling pathways, including P38 MAPK and NFκB subunits pP65 and pP50 (Table 2). Specifically, we found that female rats exhibited an attenuated microglial activation and decreased expression of several pro-inflammatory markers compared to male rats following SCI. Furthermore, our results revealed differential activation and/or subcellular localization of P38 MAPK and NFκB signaling intermediaries in microglia among sexes. These findings highlight the novel aspect of our research, which is the identification of sex-specific differences in microglial activation and signaling pathways following SCI. This is particularly significant as it not only provides insights into the underlying mechanisms contributing to sex differences in SCI outcomes but is crucial for developing targeted therapeutic interventions that can optimize recovery outcomes in male and female spinal cord injured patients.

Disparities among sexes in physiological responses to disease and injury have become an important field of investigation, and recently, studies have demonstrated the significant impact sex has on the immune system and its response to neurological injury and diseases [17], including that of SCI. During SCI, the immune system acts as a double-edged sword, playing a deleterious role in secondary tissue damage and the development of neuropathic pain, as well as facilitating debris removal, tissue remodeling, and revascularization as part of the limited endogenous response to repair the injured CNS. Herein we show that male and female microglia display significant differences in their intracellular signaling repertoire during the initial peak of the immune response after subacute SCI, with males exhibiting changes that are consistent with pro-inflammation and the potentiation of pathology, whereas females display a subdued inflammatory phenotype that is aligned more towards anti-inflammation, injury resolution, and repair. These alterations in inflammatory signaling may be indicative of sex disparities in the functioning of innate immune cells after SCI; however, further investigation is warranted to determine if the behavior of microglia is impacted by these differences. Sex disparities in the functioning of the innate immune response early after SCI would be predicted to significantly impact the types and timings of interventions that would be used in the subacute injury setting clinically for human SCI. To date, there are very limited clinical data available regarding sex-dependent disparities in spinal cord injured patients. This is primarily due to a considerably lower incidence rate of SCI among females compared to that in males, which has led to relatively fewer female subjects and a male predominance in SCI research [49]. Previously, functional evaluations conducted by Sipski et al. [3] revealed that women with motor-incomplete high tetraplegia (C1–4 levels) tended to exhibit higher functional independence measure (FIM) motor scores than male SCI patients. Nonetheless, no significant variations were noted in FIM motor scores between men and women with other levels of motor incomplete SCI. Additionally, it was observed that irrespective of the level and degree of neurologic injury, men generally exhibit better functional outcomes than women at the time of discharge from rehabilitation.

Many studies have highlighted distinctions that exist in microglial cell activation patterns between male and female rodents under various neurological conditions [9,18,50,51]. Sex-based alterations in the dynamics of the inflammatory response orchestrated by microglia after SCI may underlie the reported sex-disparities that exist in animal models and clinically [2,3,5], influencing pathological outcomes and shaping the trajectory of tissue repair and functional restoration. The present work further expands our knowledge of the sex-specific differences in the innate immune system between males and females that manifest in critical inflammatory signaling pathways during subacute SCI. While our findings did not reveal significant morphological alterations in activated microglia at the lesion center of the injured spinal cord between male and female rodents based upon the characteristic cell markers employed, an increased trend in microglial cell density at the injury epicenter, and thus, their numbers were observed, although the difference was not statistically significant between the sexes. However, using the stereotypical markers of pro-inflammatory (iNOS) and anti-inflammatory (ARG-1) innate immune cells [25], we found distinct sex differences in these enzymes after subacute SCI and, to a lesser degree, in naïve animals, with male microglia having a propensity for higher levels of iNOS than in females, suggestive of an inherent disparity between sexes and in how these microglia responded to injury.

A potential explanation for the reduced iNOS levels in female rodent microglia may be the influence of sex hormones, whether estrogen or testosterone, which have been ascribed the capacity to modulate iNOS in microglia [52]. Estrogen, in particular, has been reported to downregulate iNOS expression [52] and suppress pro-inflammatory cytokines [53], a mechanism that has been identified to contribute to estrogen’s neuroprotective activity [54,55,56]. In CNS injury, the neuroprotective effects of sex hormones was demonstrated in a model of TBI where intact female rats displayed greater tissue preservation than both ovariectomized females and the males [57]. Reductions in iNOS by estrogen are thought to be mediated through the hormone’s interference with NFκB signaling, the master transcriptional regulator of inflammation [58]. However, despite the fact that numerous studies have reported on the neuroprotective action of estrogen, its immunomodulatory activity remains an unresolved paradox [56], with estrogen eliciting either anti- or pro-inflammatory effects depending upon the nature of the immune stimulus, the type of immune response, the pathological microenvironment, and the therapeutic window or dose of estrogen administered [59]. Further experiments with measurement of the level of sex hormones during the subacute phase of SCI may permit the determination of whether a correlation exists in the observed neuroinflammatory response between male and female rodents post SCI. Additionally, future studies employing rats subjected to ovariectomy, castration, or hormone supplementation would also help to determine the extent to which the different sex hormones influence the reactive state of microglia post-SCI. This would confirm whether the attenuated inflammatory response observed in female rats with SCI can be attributed to estrogen’s influence or is produced through mechanisms that are independent from sex hormones. Improved understanding, however, of the role that the innate immune system plays in the neuroprotective action of estrogen after neurotrauma [54,55] could permit the development of more effective approaches to limiting tissue damage after injury.

The increase in ARG-1 expression in female innate immune cells after SCI would, similar to the reduced iNOS, be expected to contribute to a more favorable, dampened inflammatory environment. Elevated ARG-1 is the primary marker of the anti-inflammatory microglial phenotype, and microglia expressing ARG-1 have been implicated in promoting tissue repair and regeneration [60]. ARG-1 competes with iNOS for the common substrate arginine, reducing the amount of nitric oxide (NO) production in microglia. NO consequently drives the extent of the neuroinflammatory response [61]. We have previously shown that the regulation of iNOS and ARG-1 are inversely linked in microglia under conditions that induce pro-inflammation or conversely reverse the pro-inflammatory phenotype [25]. Estrogen has also been implicated in regulating ARG-1 expression in microglia and this action could potentially be involved in the hormone’s neuroprotective capacity in females [61]. In this study, we opted to concentrate on iNOS and ARG1 expression to specifically probe the polarization states of microglia and macrophages and their potential role in sex differences in neuroinflammatory responses. Future studies involving the comparative evaluation of the expression levels of pro-inflammatory cytokines such as TNFα, IL-1β, and IL-6 and anti-inflammatory cytokines like IL-4 and IL-10 will be necessary to determine the role that specific cytokines play in orchestrating the identified sex related differences observed in the immune response as well as their respective contributions to sex differences in the extent of secondary tissue damage or neurorepair following SCI [1]

After SCI, both male and female innate immune cells exhibited pronounced upregulation of CD86, though the increase in females was significantly greater. CD86 is considered one of the characteristic pro-inflammatory markers due to its association with antigen-presenting cell activity and the modulation of immune responses [8,47,62,63,64]. CD86 is typically elevated in activated microglia and macrophages [65,66], being important for their interactions with T cells to prime them during pathological conditions. The recent research, however, has identified CD86 in innate immune cells as a marker of both pro- and anti-inflammatory phenotypes. CD86 expression in ARG-1 immunoreactive microglia is associated with the alternative type II activation state of microglia [67,68], and it could be this phenotypic state that predominates in females compared to males in subacute SCI. Microglia in the alternative type II activation state have been shown to constitute a transitional deactivating phenotype that is associated with an immunoregulatory role and expresses anti-inflammatory mediators like IL-10 in addition to the pro-inflammatory cytokines and, therefore, have demonstrated dual roles, exhibiting both protective and pathogenic effects [67,69]. Microglia with a concurrent dual activation state have been linked to the attenuation of the inflammatory response in spinal cord injury (SCI) and myocardial ischemic injury, thereby contributing to the recovery from these injuries [64,70,71]. In that study, microglial CD86 was shown to be significantly attenuated by estrogen pretreatment [53,72]. Conversely, our results indicate an increased expression of CD86 in female microglia post-SCI. Further studies using microglia specific CD86 knockout/inhibition or hormone removal/supplementation will be required to gain insight into why female microglia exhibit increased CD86 expression. Additionally, it would be important to determine if effects of this increased CD86 expression in female microglia post-SCI modulates additional immune checkpoints. This could aid in the elucidation of whether the observed upregulation in CD86 is part of a broader shift in immune signaling pathways such as T cell activation and proliferation. Exploring this avenue following inhibition or genetic depletion of CD86 would help establish sex-dependent relevance in shaping the adaptive immune response post-SCI.

In addition to iNOS, TLR4 was also higher in male microglia following SCI. TLR4 is a transmembrane protein and pivotal component of the innate immune system that is involved in the recognition of damage-associated molecular patterns (DAMPs) at the initiation of the inflammatory response [73]. Downstream of TLR4 is the NFκB pathway, which increases pro-inflammatory gene expression and responds to pro-inflammatory stimuli, including Lipopolysaccharide (LPS). Elevated TLR4 expression in male microglia may suggest a heightened level of immune surveillance and contribute to an amplified inflammatory response after SCI. The existence of a number of TLR4 targeted pharmaceuticals may offer an immune-targeted therapy that could be more effective in males due to the elevated levels of TLR4. Conversely, heightened TLR4 could also imply a greater resistance to inflammatory stimuli in males. Enhanced TLR4 signaling has been correlated with a persistent depression-like behavior in female mice that is not present in males with microglia-selective genetic ablation of TLR4, abrogating this depression-like behavior to baseline levels in female mice [26]. Similarly, a correlation has been observed between microglial TLR4 and Alzheimer’s pathogenesis, which was more pronounced in females than males [74]. In a model of SCI, however, lower induction of TLR4 did not correlate with improvements in behavioral and histopathological outcomes [1]. Further exploration of the role of TLR4 in initiating pro-inflammatory signaling using inhibitor or knockdown strategies will be required. The results from our studies show enhanced expression of TLR4 in males concurrently with downstream pP65 NF-κB activation, which are both attenuated in female microglia. Future studies employing selective inhibitor or genetic deletion of the TLR4 receptor in male microglia will confirm if elevated TLR4 levels are a key contributory factor towards mediating the heightened inflammatory response, increased tissue damage or reduced functional outcome in male SCI rodents.

CD68, or macrosialin, a lysosomal glycoprotein expressed highly in phagocytes and a characteristic activation marker for cells of the innate immune system [75] exhibited a sex disparity in expression after subacute SCI. CD68 was greater in female microglia compared to males, suggestive of a more robust phagocytic response, and along with the observed changes in other phenotypic markers, supports the existence of a larger subpopulation in females after SCI that resume an alternative type II activated state. Increased phagocytic activity has been shown to play important roles in tissue remodeling and endogenous repair during neurological injury and disease by ensuring the removal of debris, myelin recycling, and pruning of aberrant connections. Therefore, the increased phagocytic ability of female microglia is likely to permit a faster clearance of cellular debris and the resolution of inflammation that are prerequisites for the subsequent repair phase. Studies have reported that both age and sex have significant impact on the phagocytic activity of microglia [76], with an example being the female hormone 17β-estradiol that has been shown to significantly enhance the phagocytic clearance of apoptotic cells [75]. However, TLR signaling is known to play a critical role in orchestrating the phagocytic gene program in macrophages for efficient clearance of inhibitory myelin fragments [77], and TLR4 was lower in females compared to males in this investigation. To reconcile this difference, other members of the TLR family, aside from TLR4, may be involved in directing the more robust phagocytic signaling observed in female microglia after SCI. Elucidation of these putative TLRs and functional assessments of disparities in phagocytosis by microglia derived from different sexes warrants further investigation and may shed light on novel mechanisms underlying the superior recovery of females over males after SCI [1,4,7].

The MAPK P38, which regulates cellular responses to stress and inflammation, especially in macrophages [30,78], also exhibited differences in phosphorylation levels among sexes subacutely after SCI. In macrophages the phosphorylation of P38 MAPK^Thr180/Tyr182^ is critical in triggering inflammatory responses [78], inducing the expression of pro-inflammatory mediators including TNFα, IL-6, and cyclooxygenase-2 (COX-2) [30,78,79], and when P38 is blocked, the transcriptional activity of NFκB is attenuated [80]. The role of P38 in inflammation has been ascribed to its α/β isoforms [28]. On the other hand, P38 has also been implicated in the resolution of inflammation [81] by altering the response of cells including macrophages [30], with studies demonstrating that the inhibition of P38 reduces levels of the anti-inflammatory cytokine IL-10 [82]. Therefore the role of P38 in inflammation remains controversial. In the present study, female innate immune cells displayed much higher levels of P38 phosphorylation than males after SCI, and when examined in the context of the other signaling differences, this suggests that increased P38 in these cells was associated with anti-inflammatory activities. The prior work examining the anti-inflammatory actions associated with the phosphorylation of P38α have suggested that the downstream kinases MSK1/2 and subsequent phosphorylation of the transcription factor CREB and histone H3 are involved in the positive regulation of the anti-inflammatory gene program, including the expression of IL10 [83]. Furthermore, P38 plays a critical role in the induction of the alternative activation of peritoneal macrophages by IL-4 [84] in that inhibitors of P38 block IL-4 triggered phosphorylation of STAT-6 and Akt, as well as activation of anti-inflammatory markers [84]. Examining if these signaling intermediaries show sex differences in expression or phosphorylation after SCI that are in agreement with the observed P38 disparity would further substantiate this anti-inflammatory role after SCI. Future investigations using microglia-specific targeted knockout of P38 or use of P38 -selective inhibitors in animals across sexes may allow further elucidation as to the involvement of P38 signaling molecules in the differential neuroinflammatory responses of males and females after SCI.

Another notable sex disparity observed in innate immune cells after subacute SCI was in the phosphorylation and subcellular localization of the transcription factor NFκB, as well as the kinases that are responsible for these protein modifications, the IκB kinases (IKK). NFκB is the master transcriptional regulator of inflammation and is canonically activated in response to pro-inflammatory stimuli, including neurotrauma [85]. Activation results in the release of p65 and p50 subunits from the inhibitory protein IκBα complex and allows translocation of p65:p50 NFκB dimers to the nucleus, where they bind and transactivate the promoters of pro-inflammatory genes to induce expression [86]. In this study, we analyzed key components of the NFκB signal transduction pathway including (**1**) IκB kinase (IKK), which is a complex of two catalytic subunits, IKKά and IKKβ, that have opposing roles in regulating inflammation, with IKKβ mediating NFκB activation and pro-inflammation, whereas IKKά suppresses NFκB by causing turnover or removal of NFκB subunits from pro-inflammatory gene promoters [87]; (**2**) IκBα, the most potent inhibitor of the NFκB signaling pathway, sequesters NFκB dimers in the cytoplasm in an inactive state and, if knocked out, leads to sustained NFκB activation, severe inflammation, and postnatal death [88]. Site-specific phosphorylation of IκBα protein at positions Ser 32 and Ser 36 promotes polyubiquitination and subsequent proteasomal degradation that results in the release of NFκB dimers; (**3**) the NFκB subunit p65 (RelA), which acts as the central mediator of pro-inflammatory gene induction [31] and (**4**) the NFκB subunit p50 (NF-kB1), which functions as a transcriptional regulator according to its dimerization partnering, acting as a positive regulator when bound to subunits RelA, c-Rel, and RelB or transcriptional repressor when forming p50 homodimers [89].

Among the most profound sex disparity observed in NFκB signaling was the subcellular localization of serine-337 phosphorylated p65, which was predominantly nuclear in male innate immune cells and conversely largely cytoplasmic in females. With the translocation of phosphorylated p65 to the nucleus being the primary inducer of the pro-inflammatory gene expression program, this disparity would suggest a much more robust pro-inflammatory response occurred in males after SCI compared to females and this may be the key determinant of why increased tissue preservation and reduced neurological dysfunction is observed in females after SCI [1]. In addition to this difference, females also exhibited elevated levels of IKKα compared to males, which, through its anti-inflammatory properties, would prevent the nuclear accumulation of NFκB subunits by accelerating their degradation and removal from pro-inflammatory gene promoters. Studies have demonstrated that the inactivation or the deletion of IKKα in mice produces increased inflammation [87]. The examination of IκBα, which, in the nucleus, can both inhibit NFκB/DNA interactions, as well as actively transport p65 back to the cytoplasm to regulate NFκB-dependent transcription [90,91], showed that levels were higher in male innate immune cells after SCI than females. This finding may suggest that levels of IκBα increased as a negative feedback mechanism in compensating to dampen the increased nuclear translocation of p65 after SCI in males. However, further investigation is required to provide compelling evidence supporting the role that nuclear IkBα is playing in microglia after SCI. Examination of the other NFκB subunit revealed that females had higher levels of phosphorylated, nuclear p50 after SCI compared to males. In both conjunction with and in opposition to p65, the p50 subunit plays a central role in coordinating inflammatory responses in microglia according to its dimerization [92]. Hetero- and homodimers of p50 translocate to the nucleus via their nuclear localization sequence (NLS), binding to the promoters of genes involved in inflammation [93]. However, unlike p65, the p50 subunit lacks a transactivation domain and cannot stimulate transcription without dimerization to RelA (p65), c-Rel, or RelB [92]. Therefore, homodimers of p50 act as transcriptional repressors of pro-inflammatory genes [93], a role further substantiated by the enhanced pro-inflammatory responses observed in p50-deficient mice [94], such as increased expression of TNF-α and IL-12 [95]. The serine-337 phosphorylation of p50 has been shown to enhance the DNA binding of p50 [96] and, thus, increase its ability to mediate the activation or repression of gene expression according to its dimer partner. In females, the significantly higher levels of nuclear p50 and concurrent reduction in nuclear p65 compared to males would suggest that after SCI, the NFκB signal transduction pathway would favor pro-inflammatory gene repression in females with the converse occurring in males. However, changes in other Rel subunits, which were not investigated in this study, may have occurred that would have permitted the formation of p50 heterodimers and left pro-inflammatory gene induction intact after SCI and, thus, further examination is warranted. With HDAC1 forming a repressor complex with p50 homodimers on DNA to repress inflammation-related genes including Ccl2, Cxcl10, Gm-CSF, and Mmp-13 [97], it would also be important to investigate the sex differences in HDAC-1 levels after SCI that may shed additional light on the functionality of the increased phosphorylated, nuclear p50 observed in females.

Herein it is shown that activated microglia exhibit many sex differences in the expression, phosphorylation, and subcellular localization of key signaling intermediaries associated with pro-inflammation, or the inhibition thereof, subacutely following SCI. The most profound of these differences was the subdued NFκB signaling and greater alternative activated subpopulation in females compared to males after SCI. These differences may play an important role in the previously reported improved tissue preservation and functional recovery observed in female rodents compared to males after SCI [1,4]. It would be interesting to evaluate the extent of correlation between the attenuated reactive state of female microglia following SCI to axonal regeneration, synaptic plasticity, and functional recovery. Employing gene knockdown or overexpression strategies using siRNA mediated inhibition, CRISPR-Cas9 gene editing, or viral-vector-mediated gene delivery to modulate the expression of key proteins in the NFκB signaling pathway, selectively in microglial cells, could provide direct evidence of their roles in mediating sex-specific responses to SCI.

The overall scope of our study has several limitations, notably, the absence of longitudinal investigations to monitor sex-dependent changes in the temporal alterations in key inflammatory molecules such as TLR4, CD86, pP38, and various signaling components of the NFkB pathways, both in the early and late stages post-injury that regulate microglial responses over time and their impact on SCI pathophysiology and recovery. This would allow us to identify critical windows for therapeutic intervention. Additionally, not using female rodents subjected to ovariectomy or castrated male rodents limits our understanding of how sex hormones affect microglial reactivity post-SCI and assessment of the role that specific signaling intermediaries play in functional outcomes necessitates the use of microglia-specific conditional knockout animals for TLR4, P38, and P65 or P50. While the work focuses on microglia, other immune cells including neutrophils and infiltrating macrophages also are integral to the broader immune response and may also exhibit sex-based differences after SCI that were not investigated in this study. Even though this study primarily focuses on determining the role of microglia in mediating sex differences in the neuroinflammatory response to SCI, evaluating the role of infiltrating neutrophils and macrophages using specific markers such as CD45 and Ly6C/G will be crucial to employ in the future to identify whether these peripheral immune cells contribute to the sex-dependent innate immune response following SCI. Despite the need for further and expanded investigation, we believe that the findings of our study significantly contribute to our understanding of sex-specific responses at the cellular and molecular levels after SCI. Overall, the work sheds light on the importance of considering sex as a biological variable in SCI research, emphasizing the need for clinical studies to also consider sex in the development of therapies targeting microglial activation and signaling pathways for improved outcome in SCI patients.

## 5. Conclusions

In conclusion, our study examined sex-specific differences in microglial activation and signaling pathways after SCI. We found significant disparities between male and female rats in microglial activation and the expression levels of key inflammatory signaling proteins, including P38 MAPK and NFκB subunits pP65 and pP50. Female rats exhibited reduced microglial activation and expression of pro-inflammatory markers compared to males following SCI. By elucidating sex-specific responses to SCI at the cellular and molecular levels, our study provides valuable insights into the heterogeneity of SCI pathology and recovery. These findings highlight the novelty of our research, revealing sex-specific discrepancies in microglial responses to SCI. These insights underscore the importance of considering sex as a biological variable in the development of targeted therapeutic interventions for SCI and for optimizing recovery outcomes. Overall, our findings enhance our understanding of SCI pathology and recovery, emphasizing the need for personalized treatment approaches that address sex-specific differences.

## Figures and Tables

**Table 1:** Primary antibodies used for flow cytometry and immunohistochemistry.

Antibodies Used for Flow Cytometry				
Primary Antibody	Host	Manufacturer	Catalog Number	Dilution
Anti-Rat CD11b:Pacific Blue	Mouse	Bio-Rad (Hercules, CA, USA)	MCA275PB	1:50
PE anti-mouse CX3CR1	Mouse	BioLegend (San Diego, CA, USA)	149006	1:100
Human/Mouse anti-Arginase 1-APC	Sheep	R&D Systems (Minneapolis, MN, USA)	IC5868A	1:10
Anti-mouse iNOS-Alexa Fluor 488	Rat	Invitrogen (Waltham, MA, USA)	53-5920-82	60 µg/mL
Anti-rat CD163:FITC	Mouse	Bio-rad	MCA342F	1:10
Anti- MMR/CD206-APC conjugated	Goat	R&D Systems	FAB2535A	1:10
Anti-rat CD68-Alexa Fluor 647	Mouse	Bio-rad	MCA341A647	1:10
FITC anti-rat CD86	Mouse	BioLegend	200305	6.25 µg/mL
FITC anti-rat CD38	Mouse	BioLegend	250503	1:50
Anti-rat CD38 (14.27) eFluor660	Mouse	Invitrogen	50-0380-80	2.5 µg/mL
pAb anti-EGR2 [FITC]	Rabbit	Novus Biologicals (Centennial, CO, USA)	NB110-59723F	1:10
Anti-NOS2 (C-11) FITC	Mouse	Santa Cruz Biotechnology	SC-7271 FITC	10 µg/mL
SIGLEC H Monoclonal Antibody (eBio440c), PE	Rat	Fisher Scientific (Hampton, NH, USA)	50-103-25	1.25 μg/mL
**Antibodies Used for Immunohistochemistry**				
**Primary Antibody**	**Host**	**Company**	**Catalog Number**	**Dilution**
Anti-Arginase 1	Rabbit	Genetex (Irvine, CA, USA)	CTX109242	1:100
Anti-MRC1	Rabbit	Sigma-Aldrich (St. Louis, MO, USA)	HPA045134	1:100
anti-Phospho-p38 MAPK (Thr180/Tyr182) (D3F9)	Rabbit	Cell Signaling Technology (Danvers, MA, USA)	4511S	1:400
Phospho-p38 MAPK (Thr180/Tyr182)	Rabbit	Cell Signaling Technology	9211	1:200
Anti-NOS2 (C-11) FITC	Mouse	Santa Cruz Biotechnology	sc-7271	1:50
Anti-TLR4	Mouse	Abcam (Waltham, MA, USA)	ab22048	1:100
Anti-Iba1	Goat	Abcam	ab5076	1:500
Anti Rat CD68, clone ED1	Mouse	Bio-rad	MCA341R	1:100
Anti-rat CD163	Rabbit	Bio-rad	MCA342R	1:100
Phospho-IKKα (Ser176, Ser180)	Rabbit	ThermoFisher Scientific (Waltham, MA, USA)	44-714	1:200
Phospho-IKKβ (Tyr188) Polyclonal	Rabbit	Invitrogen	PA5-104693	1:100
Anti-IL-1 beta	Rabbit	Abcam	ab2105	1:100
Anti-IL-1 beta (E7-2-hIL1β)	Mouse	Santa Cruz Biotechnology	sc-32294	1:100
Anti Iba1, Rabbit (for ICC)	Rabbit	Wako/Fuji (Richmond, VA, USA)	019-19741	1:1000
Chicken Polyclonal to Iba1	Chicken	EnCor Biotechnology (Gainesville, FL, USA)	CPCA-Iba1	1:1000
Goat pAb to Iba1	Goat	Abcam	ab5076	1:500

**Table 2:** Sex-dependent differences in pro- and anti-inflammatory factors in innate immune cells within the injured spinal cord after subacute SCI.

Pro-Inflammatory	Male	Female
**iNOS**	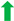	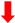
**CD86**	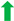	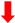
**TLR4**	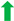	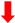
**pP38 MAPK**	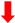	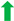
**IKKβ**	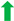	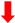
**IKBα**	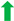	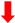
**NFκB pp65**	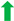	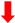
**Anti-Inflammatory**	**Male**	**Female**
**ARG1**	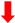	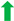
**CD68**	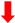	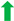
**MRC1/CD206**	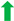	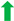
**pP38 MAPK**	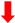	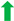
**IKKα**	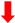	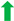
**NFκB pp50**	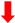	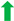

Female microglia show an attenuated pro-inflammatory phenotype and an enhanced expression of anti-inflammatory factors associated with neuro-protection and -repair after subacute SCI within the injured spinal cord compared to males. 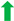 indicates increased expression level and 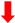 suggests decreased levels of target proteins.

## Data Availability

The data that support the findings of this study will be made available from the corresponding author upon reasonable request.
